# Determination of the Prevalence of Triazole Resistance in Environmental *Aspergillus fumigatus* Strains Isolated in South Wales, UK

**DOI:** 10.3389/fmicb.2018.01395

**Published:** 2018-06-26

**Authors:** Alexandra Tsitsopoulou, Raquel Posso, Lorna Vale, Scarlett Bebb, Elizabeth Johnson, P. L. White

**Affiliations:** ^1^Regional Mycology Reference Laboratory, Public Health Wales, Microbiology Cardiff, Cardiff, United Kingdom; ^2^National Mycology Reference Laboratory, Public Health England, Bristol, United Kingdom

**Keywords:** *A. fumigatus*, azole resistance, TR34, L98H, environmental prevalence

## Abstract

**Background/Objectives:** Azole resistance in *Aspergillus fumigatus* associated with the TR34/L98H mutations in the *cyp51A* gene have been increasingly reported. Determining the environmental resistance rate has been deemed important when considering front-line therapy for invasive aspergillosis. The aim of the study was to determine prevalence of azole resistance in environmental *A. fumigatus* isolates across South Wales.

**Methods:** Over 5 months in 2015, 513 *A. fumigatus* isolates were cultured from 671 soil and 44 air samples and were screened for azole resistance using VIPcheck™ agar plates containing itraconazole, voriconazole and posaconazole. Resistance was confirmed by the CLSI M38-A2 methodology. The mechanism of resistance was investigated using the PathoNostics AsperGenius® Assay.

**Results:** Screening by VIPcheck™ plate identified azole-resistance in 30 isolates, most of which (28/30) harbored the TR34/L98H mutation, generating a prevalence of 6.0%. Twenty-five isolates had a MIC of ≥2 mg/L with itraconazole, 23 isolates had a MIC of ≥2 mg/L with voriconazole and seven isolates had a MIC ≥0.25 mg/L with posaconazole. All isolates deemed resistant by VIPcheck™ plates were resistant to at least one azole by reference methodology.

**Conclusions:** There is significant environmental azole resistance (6%) in South Wales, in close proximity to patients susceptible to aspergillosis. Given this environmental reservoir, azole resistance should be routinely screened for in clinical practice and environmental monitoring continued.

## Introduction

*Aspergillus fumigatus* causes a variety of diseases in humans ranging from allergic bronchopulmonary aspergillosis (ABPA), through chronic pulmonary aspergillosis and aspergilloma, to invasive aspergillosis (IA) (Latge, 1999). Triazole therapy has become the established treatment for these infections, based on their proven efficacy and ease of administration, but patients often require prolonged therapy (Patterson et al., 2016). Monitoring for resistance to these drugs is therefore imperative, as treatment failure, or delayed appropriate therapy will increase mortality.

Current data suggests that resistance can develop by two routes; *in situ* within the lungs of a chronically infected patient as a result of prolonged exposure, or by acquisition of a resistant *A. fumigatus* strain from the environment, potentially driven by agricultural use of azole compounds (Verweij et al., 2009; Denning et al., 2013). The prevalence of azole resistant *A. fumigatus* in most countries is still not clearly determined as routine resistance testing is not common practice. A recent report from the European Centre for Disease Prevention and Control acknowledges that resistant *A. fumigatus* strains are spreading in many European countries and efforts to monitor resistance rates need to be undertaken (Risk assessment on the impact of environmental usage of triazoles on the development and spread of resistance to medical triazoles in *Aspergillus* species., 2013). Environmental surveillance studies of azole resistance have been undertaken in many countries including Italy, Austria, India, Thailand and the USA (Mortensen et al., 2010; Chowdhary, 2012; Prigitano et al., 2014; Hurst et al., 2017; Tangwattanachuleeporn, 2017). Resistant isolates harboring either TR34/L98H or TR46/Y121F/T289A mutations have been found in environmental and clinical samples from several countries, raising concern that azole resistance could become a global public health threat with fungal spores able to disperse great distances on air currents (Bader, 2015; Verweij et al., 2015a).

Data is also lacking in the UK. Research performed by the Centre for Aspergillosis in Manchester showed resistance rates in clinical isolates collected between 1992 and 2007 was approximately 6%. A great variability of mutations in the *cyp51*A gene (18 in total, including two TR34/L98H mutations) was found with no prevalent mutation, indicating a clinically driven route of resistance (Howard et al., 2009). In the corresponding study of several hundred environmental isolates, from both rural and urban areas, only one isolate with azole resistance driven by the TR34/L98H mutation was found, originating from the rural environment (Bromley et al., 2014).

The aim of the present study was to investigate the prevalence of azole resistance in environmental *A. fumigatus* isolates, collected primarily in South Wales, and to establish an appropriate method for screening for azole resistance. One such method which utilizes agar with set concentrations of three different azoles (VIPcheck™ plates) was devised in the Netherlands and showed promising results (Van der Linden et al., 2009; Buil et al., 2017). It is a cheap, fast and simple method requiring no specialist skill or equipment to screen a high number of specimens. If accurate it would be ideal as a screening tool suitable for use outside of reference settings.

## Materials and methods

### Environmental sampling

#### Soil samples

**Figure 1:**
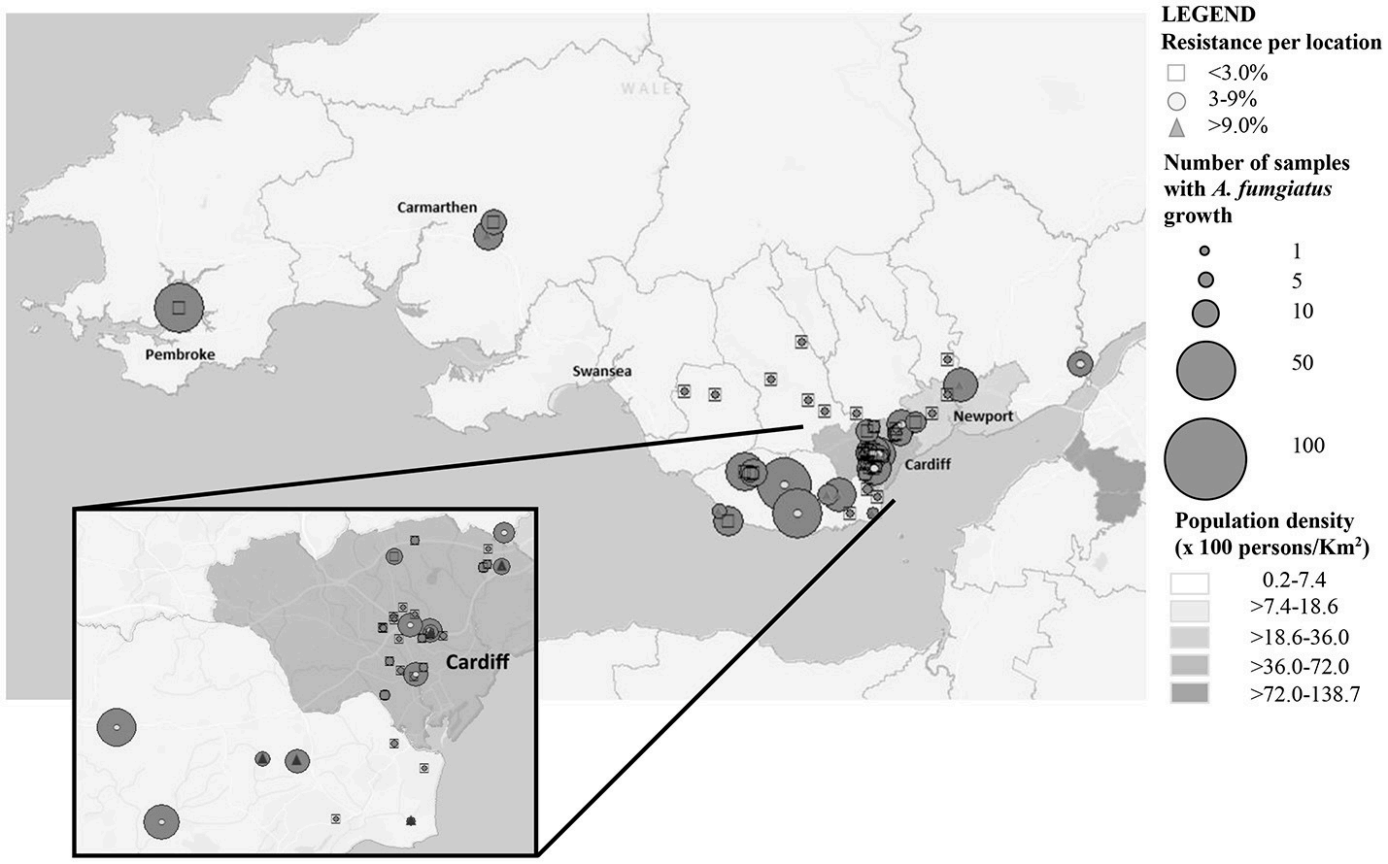
Soil sampling distribution and intensity across South Wales.

From June to November 2015, 671 soil samples were collected from urban and rural locations in South Wales (Table 1 and Figure 1). The samples were treated as previously described to optimize recovery of *A. fumigatus*, with minor modifications (Snelders et al., 2009). Briefly, 2 g of soil or compost was dissolved in 8 mL of sterile distilled water with 1% Tween 20 (Sigma, Haverhill, UK). After mixing it was left to sediment for 30–60 min at room temperature and 100 μL of the supernatant was used to inoculate two plates of Sabouraud dextrose agar supplemented with chloramphenicol (E&O Laboratories Limited, Bonnybridge, UK). The plates were incubated at 37° and 42°C to maximize the selective yield of *A. fumigatus* isolates, and examined after 48 h of incubation. *A. fumigatus* isolates were phenotypically identified by observation of macroscopic and microscopic morphology.

**Table 1:** Soil sampling areas and rates of *A. fumigatus* recovery, with azole resistance determined by VIPcheck™ plate.

**Sampling site**	**Samples collected (*n*)**	**Number of *A. fumigatus* isolates recovered (%)**	**Azole susceptibility**^**a**^			**Resistance rates (95% CI)**
			**Resistant (*n*)**	**Indeterminate (*n*)**	**Sensitive (*n*)**	
**AGRICULTURE**						
Overall	419	288 (68.7)	15	49	224	5.2% (3.2–8.4)
Beans fields	37	29 (78.4)	1	8	20	3.4% (0.6–17.2)
Cereal fields	171	120 (70.2)	4	21	95	3.3% (1.3–8.3)
Clover fields	11	6 (54.5)	0	0	6	0% (0.0–3.9)
Compost	6	4 (66.6)	0	1	3	0% (0.0–4.9)
Corn fields	37	26 (70.3)	4	7	15	15.4% (6.2–33.5)
Environmental strips	2	2 (100)	0	0	2	0% (0.0–6.6)
Grass fields	7	2 (28.6)	1	0	1	50.0% (9.5–90.6)
Potato fields	83	46 (55.4)	1	5	40	2.2% (0.4–11.3)
Rapeseed fields	65	53 (81.5)	4	7	42	7.5% (3.0–17.9)
**NONPUBLIC AREAS**						
Overall	35	28 (80.0)	0	9	19	0% (0.0–11.4)
Private allotments	2	2 (100)	0	1	1	0% (0.0–65.8)
Private gardens	33	26 (78.8)	0	8	18	0% (0.0–12.1)
**HORTICULTURAL NURSERY**						
Compost	1	1 (100)	0	0	1	0% (0.0–79.4)
**PUBLIC AREAS**						
Overall	216	179 (91.2)	15	32	132	8.4% (5.1–13.4)
Botanical gardens	31	27 (87.1)	7	7	13	25.9% (13.2–44.7)
Compost	5	5 (100)	0	1	4	0% (0.0–43.5)
Hospital grounds	26	24 (92.3)	1	5	18	4.2% (0.7–20.2)
Parks	35	22 (62.9)	0	0	22	0% (0.0–14.9)
Public gardens	119	101 (84.9)	7	19	75	6.9% (3.4–13.6)
(Hospital gardens)^b^	(27)	[22 (81.5)]	(3)	(6)	(13)	[13.6% (4.8–33.3)]
COMBINED TOTAL	671	496 (73.9)	30	90	376	6.0% (4.3–8.5)

a Resistance determined by confluent growth in the presence of at least one azole antifungal drug.

b *Data for Hospital Gardens is accounted for in the data for Public gardens and to avoid duplication has not been included separately in the overall counts*.

#### Air samples

During the same time period 44 air samples were collected from clinical sites in three major hospitals in the Cardiff and Newport area. Air samples were collected with single-headed SAS super 100 Air sampler (Cherwell Laboratories, Bicester, UK). Five hundred liters of air was directed onto each contact Sabouraud plate supplemented with chloramphenicol. The plates were then incubated and any *A. fumigatus* isolates were confirmed as previously described.

### Screening for azole resistance

**Figure 2:**
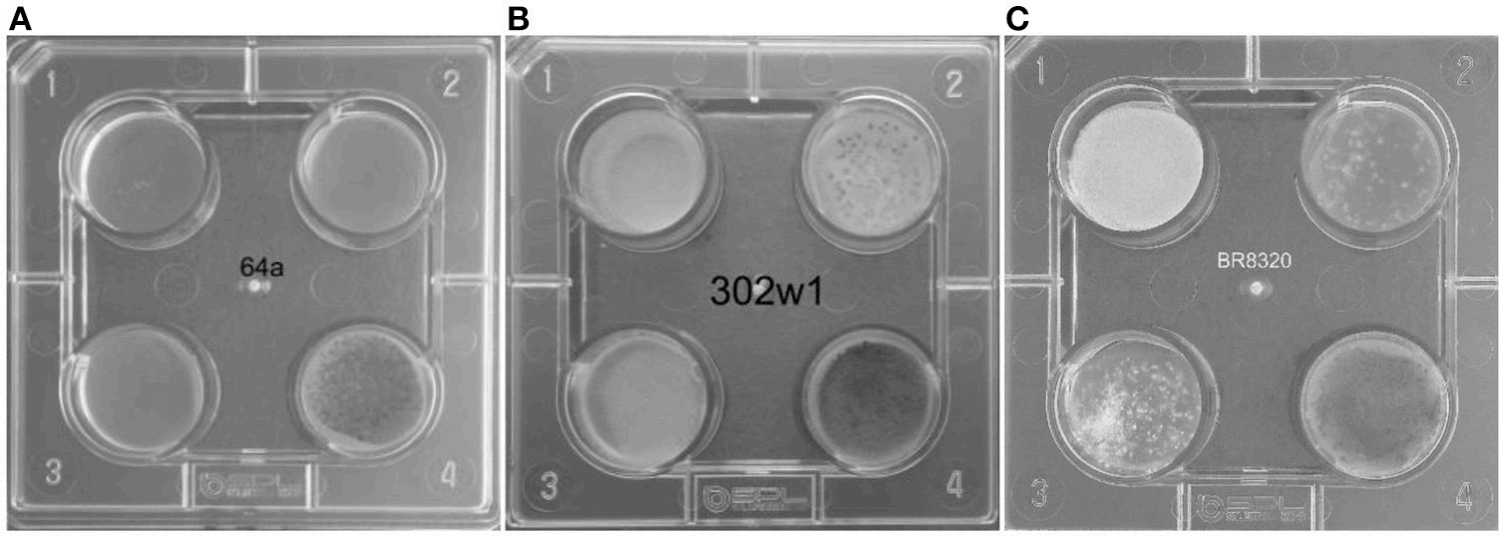
VIPcheck™ plates containing itraconazole at concentration of 4 mg/l (Well 1; top left), voriconazole at a concentration of 2 mg/l (Well 2; top right), posaconazole at a concentration of 0.5 mg/l (Well 3; bottom left) and a control well containing only RPMI agar (Well 4; bottom right) for **(A)** a fully azole sensitive environmental strain of *A. fumigatus* (64a); **(B)** a pan-azole resistant environmental strain of *A. fumigatus* (302w1) and **(C)** an itraconazole resistant environmental strain of *A. fumigatus* (BR8320) showing sporadic growth in the presence of voriconazole and posaconazole.

Primary screening for azole resistance was performed using the commercially available VIPcheck™ plates containing azoles (Balis Laboratorium VOF, Leeuwen, Netherlands). The four well screening plates contained RPMI-1640 agar medium supplemented with 4 mg/L itraconazole, 2 mg/L voriconazole and 0.5 mg/L posaconazole and a fourth control well (Figure 2). Using a dry cotton wool swab, conidia from fresh sporulating colonies were suspended in sterile water to produce a suspension equivalent to 0.5–2 McFarland standard. All the wells were inoculated with 25 μL of the suspension. To enhance efficiency and reduce costs of screening, conidia from more than one colony were combined to prepare the suspensions. However, when multi-azole resistance was noted each well exhibiting growth of *A. fumigatus* was individually sub-cultured and VIPcheck™ plates inoculated to determine if there was a single multi-azole resistant strain or if there were individual strains with different patterns of azole resistance. The screening plates were then incubated at 37°C for 48 h and visually inspected, with growth quantified as good/confluent (+), sporadic (±) or no growth (–). Resistance was determined by good/confluent growth on at least one azole containing well, with or without growth on the other two azole containing wells. Where confluent growth was not present on at least one azole containing well but sporadic growth was observed on at least one azole containing well the isolate was classified as indeterminate.

Any resistant isolates were subsequently confirmed by comparison to the reference CLSI broth micro-dilution (CLSI) M38-A2 method, performed at the Public Health England Mycology Reference Laboratory in Bristol. Resistance to itraconazole and voriconazole in *A. fumigatus* was determined using epidemiological cut-offs (1 mg/L) as defined in the CLSI M59 ED1E document (CLSI: M59-ED1., 2016). As an epidemiological cut-off for *A. fumigatus* to posaconazole is not available in the CLSI M59 document EUCAST antifungal clinical breakpoint (Version 8.0) of >0.25 mg/L was used (EUCAST, 2015).

### Molecular determination of the azole resistance mechanisms

All resistant strains, 30 indeterminate and 20 sensitive strains had DNA extracted from conidia by bead-beating and automated nucleic acid extraction using the EZ1 Advance XL tissue kit (Qiagen, Crawley, UK). Conidia from sporulating cultures were mechanically disrupted for 30 s using the equivalent of 20–30 μL of MagNA Lyser green ceramic beads (Roche, Burgess Hill, UK) and a Mini bead-beater (Biospec Products, Bartlesville, Oklahoma, USA). The beads were then washed with 190 μL of G2 buffer (from EZ1 Tissue kit) and the extraction was completed following the manufacturer's protocol. Nucleic acid was eluted in 100 ul, with a further incubation at 70°C for 10 min to remove excess ethanol.

Extracted DNA was subsequently tested using a commercially available AsperGenius® real-time PCR assay (PathoNostics, Maastricht, Netherlands). The AsperGenius® assay is a multiplex real-time PCR assay that can detect *A. fumigatus, A. terreus, Aspergillus* species (Chong, 2015). In a second multiplex reaction, the AsperGenius® resistance assay has the ability to detect the four mutations (TR34, L98H, Y121F, and T289A), representing the most prevalent mutations L98H/TR34 and TR46/Y121F/T289A in the *cyp*51A gene of *A. fumigatus* associated with azole resistance. The successful amplification of the region of *cyp*51A gene also confirmed the isolate to be *A. fumigatus*. PCR was performed in a 12.5 μL volume containing 5 μL AsperGenius® Resistance mastermix, 1 μL Taq polymerase, 4 μL dilution buffer and 2.5 μL DNA extract. PCR was performed on a Rotorgene Q high-resolution melt instrument (Qiagen, Crawley, UK) following the manufacturer's instructions.

### Statistical analysis

Ninety-five percent confidence intervals (95% CI) were generated for each proportionate value and, where required, two-tailed P values were calculated using Fishers exact test; (P: 0.05) to determine the significance between rates. Observed agreement and Kappa statistic between the different methods were calculated using 2 × 2 tables, with the concentration of individual azole in the VIPcheck™ plate as the defining factor. For example, for posaconazole 0.5 mg/L is present in the VIPcheck™ plate, agreement between methods was confirmed when confluent growth was observed on the VIPcheck™ plate and the MIC by the reference method was ≥0.5 mg/L, or sporadic/no growth was observed on the VIPcheck™ plate and the MIC by the reference method was <0.5 mg/L.

## Results

### Resistance rates in environmental *aspergillus fumigatus* isolates

**Figure 3:**
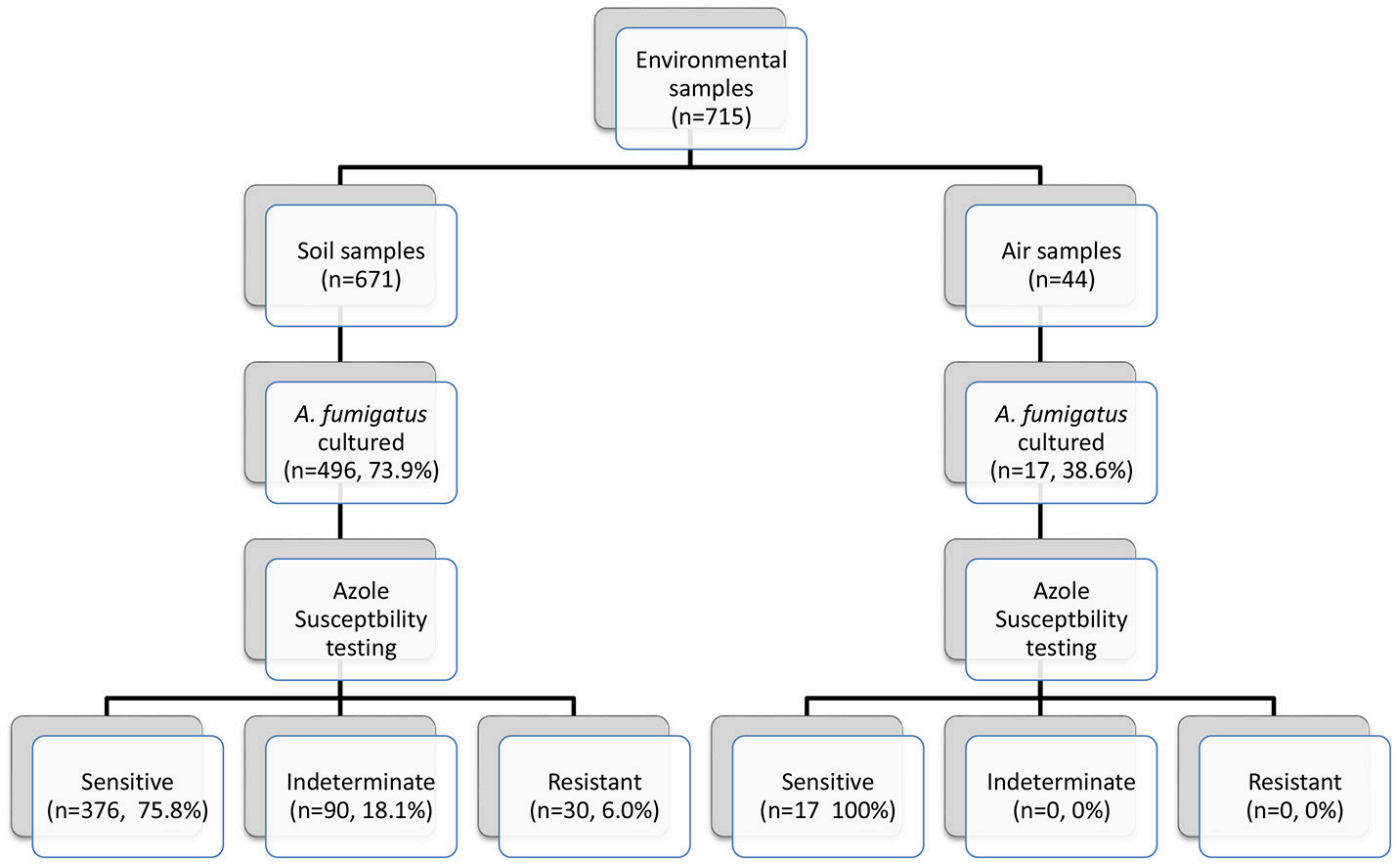
Environmental sampling scheme and breakdown of azole susceptibility testing.

Seven hundred and fifteen environmental (soil and air) samples were collected. *A. fumigatus* isolates grew in 496/671 soil samples (73.9%) and in 17/44 air samples (38.6%) (Figure 3). Thirty soil isolates showed resistance to azoles, while 376 (75.8%) soil isolates were susceptible by screening on VIPcheck™ plates. Ninety (18.1%) soil isolates demonstrated indeterminate growth in the presence of at least one azole (See Wells 2 and 3, Figure 2C). All 17 air isolates, were susceptible to azoles.

The prevalence of azole resistance across South Wales was 6.0% (95% CI: 4.3–8.5). Table 1 and Figure 1 show the sampling sites, population density, sampling intensity and location of resistant isolates across the testing region. The lowest resistance rates were found in south central regions [3.9% (95% CI: 1.7–8.8), 5/128 *A. fumigatus* isolates], while the highest rates were associated with the Gwent/Monmouthshire region (10.5% (95% CI: 4.2–24.1), 4/38 isolates). The rate of resistance in Cardiff and the surrounding rural areas was 6.8% (95% CI: 4.3–10.7, 17/249 isolates), whereas the rate in South West Wales was 5.2% (95% CI: 2.0–12.6, 4/77 isolates). The four isolates from South West England were sensitive to azole antifungal drugs. In seven locations, spread across the testing area, the rate of azole resistance was >10% (Range 11.1–44.4%) (Figure 1). The highest rate (4/9 isolates) was associated with a botanical garden in a city center park that was within one mile of a tertiary referral hospital caring for patients who would be at risk of IA. A rate of 13.6% (95% CI: 4.8–33.3, 3/22 isolates) was also associated with isolates cultured from a public garden within the grounds of a district general hospital.

The rate of azole resistance in agricultural areas was 5.2% (95% CI: 3.2–8.4), compared to 8.4% (95% CI: 5.1–13.4) in public areas, many within urban settings. The difference between rates of resistance in these settings were not statistically different (Difference: 3.2%, 95% CI: −1.4 to 8.6, *P*: 0.1799). Disturbingly, four resistant isolates were recovered from the grounds/gardens across two hospital sites (resistance rate 8.7%, 95% CI: 3.4–20.3). The rate of resistance in botanical gardens (7/27; 25.9%) was significantly greater than other areas combined (23/469; 4.9%) (Difference 21.0%, 95% CI: 8.1–39.9, *P*: 0.0005). No resistant isolates were cultured from private gardens/allotments or compost heaps, although sampling of such areas was limited. and indeterminate growth was evident (32.1%).

All 30 *A. fumigatus* isolates exhibiting azole resistance by VIPcheck™ plate screening were resistant to itraconazole, with 24 and 19 isolates exhibiting at least sporadic growth on the voriconazole and posaconazole wells. Four isolates only showed resistance to itraconazole, with two showing growth on itraconazole and sporadic growth on posaconazole, while seven showed growth on itraconazole and sporadic growth on voriconazole. Seventeen isolates potentially showed pan-azole resistance, when considering sporadic growth as significant. Resistance to itraconazole was always exhibited by confluent growth (30/30), whereas 21/24 and only 2/19 isolates showed confluent growth on voriconazole and posaconazole-containing wells, respectively.

### Confirmation of azole resistance by CLSI M38-A2 broth microdilution testing

When testing for itraconazole resistance by the reference method, 25/30 (83.3%, 95% CI: 66.4–92.7) VIPcheck™ itraconazole resistant isolates generated resistant minimum inhibitory concentrations (MIC). Twenty-one had a MIC of >16 mg/L, three with 4 mg/L and one with 2 mg/L. Of the five VIPcheck™ itraconazole resistant/CLSI susceptible isolates four had a MIC of 1 mg/L and one 0.5 mg/L. Agreement with VIPcheck™ testing was 80% (95% CI: 62.7–90.5), but it was not possible to calculate a Kappa statistic.

When testing for voriconazole resistance by the reference method 23/30 (76.7%, 95% CI: 59.1–88.2) generated resistant MIC values. Sixteen had resistant MIC of 2 mg/L and seven of 4 mg/L. Of the seven CLSI voriconazole susceptible isolates five had a MIC of 1 mg/L, one of 0.5 mg/L and one of 0.25 mg/L. When comparing voriconazole resistance as determined by the VIPcheck™ plate with the CLSI method 19 were deemed resistant by both, 5 were susceptible by both, whereas two were resistant by VIPcheck™ plate only (CLSI method MIC 1 mg/L) and four resistant by CLSI method only (MIC 2 mg/L). This generated an observed categorical agreement of 80.0% (95% CI: 62.7–90.5) and a Kappa statistic of 0.49, representing fair/good agreement.

When testing for posaconazole resistance by the CLSI method 7/30 showed resistance (23.3%, 95% CI: 11.8–40.9), six generating resistant MIC values of 0.5 mg/L and one of 1 mg/L. A further 21/30 had an intermediate MIC of 0.25 mg/L (70.0%, 95% CI: 52.1–83.3). The MIC of the two susceptible isolates were 0.12 mg/L and 0.06 mg/L, respectively. Both isolates showing confluent growth on the VIPcheck™ posaconazole well (Well 3 Figure 2B) were resistant by the CLSI method. For 17 isolates with sporadic growth on VIPcheck™ plates (Well 3 Figure 2C) 11, five and one had a MIC of 0.25, 0.5, and 0.06 mg/L, respectively. Of the 11 VIPcheck™ posaconazole susceptible isolates 10 had a MIC of 0.25 mg/L and one had a MIC of 0.12 mg/L by the CLSI method. This generated an observed categorical agreement of 83.3% (95% CI: 66.4–92.7) and a Kappa statistic of 0.38, representing fair agreement.

### Determining the molecular mechanism of azole resistance using the pathonostics aspergenius® resistance assay

**Figure 4:**
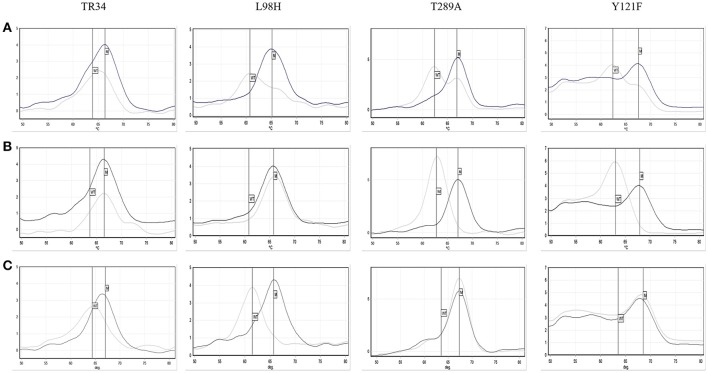
Pathonostics Aspergenius® melt-curve analysis for the TR34/L98H and T289A/Y121F mutations, with typical melting temperatures for wild-type (WT) and mutations (M) indicated by the respective lines with initial testing direct from VIPcheck™ plate showing ambiguous results **(A)**, repeat testing from VIPcheck™ plate showing the TR34/L98H mutation **(B)** and repeat testing from purity plate showing the T289A/Y121F mutation **(C)**. The control is represented by the black line and the isolate is represented by the gray line.

The AsperGenius® PCR results showed that 28/30 96.7% (95% CI: 83.3–99.4) azole resistant strains had the TR34/L98H mutations. For one isolate the results were unclear, with the presence of TR34 indicated, but both wild-type and mutation indicated for L98H and TR46/Y121F/T289A (Figure 4). Retesting a colony from the VIPcheck™ plate confirmed the presence of the TR34/L98H mutations. However, retesting a colony from a purity plate confirmed the presence of the TR46/Y121F/T289A mutations, but the TR34/L98H mutations were absent, indicating that more than one resistant strain was present. One resistant isolate did not possess any of the targeted mutations. Twenty azole-sensitive and 30 indeterminate strains did not possess any of the mutations targeted.

## Discussion

The study showed that the overall prevalence of azole resistant *A. fumigatus* in the environment is 6%, ranging from 3.9% in central regions to 10.5% in eastern regions. The resistance rate was 5.2% in agricultural areas compared to 8.4% in urban areas, including public gardens (6.9%) and hospital grounds (8.7%). One concerning discovery was a significantly greater rate of azole resistance associated with botanical gardens (25.9%). This is a marked difference when compared to the only other UK-based environmental prevalence study performed in Manchester, where environmental resistance was detected in 4/231 rural samples (prevalence of 1.7%) and no resistant isolates were found in the urban environment (Bromley et al., 2014). One explanation, are the differences in city size and local geography. Cardiff is significantly smaller than Greater Manchester and is largely surrounded by agriculture. Although there is significant agriculture around Manchester it is flanked by the Pennines and several other large towns and cities. The heavy fungicidal compound use around Cardiff potentially drives the rural resistance that easily disperses, via coastal air currents, into the urban area due to its immediate vicinity and smaller city size. PCR also confirmed an agricultural source with the vast majority of the resistant isolates (28/30) containing the TR34/L98H mutation (Snelders et al., 2009). However, in one isolate with confirmed azole resistance the mechanism of resistance has not been identified. Interestingly, it has very high MIC for (>16 mg/L) itraconazole and posaconazole (>1 mg/L), but it appears to be sensitive to voriconazole (0.12 mg/L). The exact mechanism of resistance for this isolate is being investigated by whole genome sequencing.

Of concern, is that the highest rates of resistance have been found in highly populated urban areas, where the population density of highly susceptible patients is greatest (Figure 1). Understanding local rates of environmental resistance is critical to efficient patient management and it has been proposed that the level of environmental *A. fumigatus* azole resistance should be used to guide treatment of *Aspergillus* infections (Snelders et al., 2011). It has been advocated that in areas with a high environmental azole resistance rate (designated as >10%), the use of voriconazole monotherapy for primary treatment of clinical aspergillosis should be reconsidered (Verweij et al., 2015a). The current environmental resistance rate (6%) is of concern, but opinions are divided over whether to change the primary therapy in the clinic (Verweij et al., 2015b). With the availability of highly sensitive and rapid diagnostics with the capacity to detect the most frequent mutations a strategic approach incorporating enhanced resistance screening is justified.

In this study, the VIPcheck™ plate method was assessed as a frontline screen for resistance and its accuracy corroborated by the CLSI M38-A2 reference method. All isolates with resistance determined using the VIPcheck™ plate were resistant to itraconazole and 83% of these were confirmed by the CLSI method, although one of the VIPcheck™ itraconazole resistant isolates had a CLSI MIC of 2 mg/L below the concentration in the plate. Eighty percent (4/5) that were itraconazole sensitive by the CLSI method had MIC values on the epidemiological cut-off (1 mg/L). Similar concordance (80%) was obtained for voriconazole resistance with most discrepancies showing MIC values around the cut-off. Of the three isolates showing sporadic growth on the voriconazole well two had a resistant MIC (2 mg/L). For posaconazole categorical agreement between the VIPcheck™ and CLSI method was also 83%. Five isolates that were resistant by the CLSI method (MIC: 0.5 mg/L) showed sporadic growth on VIPcheck™ plates. Conversely, 12 isolates deemed indeterminate by the CLSI method (MIC: 0.25 mg/L) showed sporadic growth on the VIPcheck™ plate, and only a single isolate with sporadic growth was genuinely susceptible (MIC: 0.06 mg/L). The presence of sporadic growth on the posaconazole well accompanied with confluent growth in at least one of the other azole containing wells likely represents a raised MIC, but also highlights the difficulty in interpreting MIC values when a gray zone exists (posaconazole breakpoints: *S* <0.125 mg/L; *R* >0.25 mg/L). With the VIPcheck™ plates containing posaconazole at a concentration of 0.5 mg/L, isolates with intermediate resistance may fail to grow. Nevertheless, it is widely accepted that itraconazole is a good marker for resistance derived from mutations in the *cyp51A* gene (Stensvold et al., 2012). All isolates that were resistant to itraconazole by VIPcheck™ plate were resistant to at least one azole by the CLSI method. Over 90% of resistant isolates had the TR34/L98H mutation and this is said to confer pan-azole resistance (Stensvold et al., 2012). Only two and seven of the isolates were shown to be pan-azole resistant by the VIPcheck™ and CLSI methods, respectively. However, a further 17 isolates would have been considered pan-azole resistant on VIPcheck™ plates if sporadic growth on posaconazole was considered significant and 21 isolates had a CLSI MIC to posaconazole of 0.25 mg/L. Next generation sequencing is currently being performed on these isolates to confirm the presence of the TR34/L98H mutation and to possibly identify other mutations.

There were 90 isolates that were deemed indeterminate as sporadic growth was observed in at least one of the azole wells of the VIPcheck™ plate, but in the absence of any confluent growth. A subset of 30 isolates was tested by the pathonostics assay but no mutations (TR34/L98H or TR46/Y121F/T289A) were present. Unfortunately, due to cost limitations MIC testing and next generation sequencing of these isolates has not been performed to confirm or refute presence of resistance.

The major limitation of the study is that as VIPcheck™ plates were used for the front-line screen it is possible that resistant strains may have been detected using other methods, although a small subset of sensitive isolates that were subjected to confirmatory susceptibility testing remained sensitive to all azoles (results not shown). In addition, as confirmatory susceptibility testing was focused mainly on resistant isolates observed agreement is likely to be under-estimated. A further limitation coincides with the screening mechanism employed, which enhanced the opportunity for detection of resistance but in doing so also allows for more than one resistant strain to be present.

This study suggests that screening for azole resistance in environmental *A. fumigatus* isolates is needed for all centers caring for patients at risk of aspergillosis. The overall rate of environmental azole resistance (6%) dictates that monitoring for resistant disease and environmental surveillance is essential, but this study shows that resistance rates can vary significantly between geographically close regions and it is essential that a single resistance rate is not universally applied. The VIPcheck™ plates provided a simplistic and cost effective method for determining azole resistance based on the itraconazole susceptibility profile. The level of the TR34/L98H mutation was high, indicating that the primary source of azole resistant *Aspergillus* in the region will be attributed to agricultural azole use. The AsperGenius® assay provides a robust means for screening for the most common resistance mechanisms. All resistant isolates will be subjected to next generation sequencing as part of an international study into azole resistance in *A. fumigatus* and this will provide essential and evolutionary information, confirm and identify mechanisms of resistance and possibly establish links between clinical and environmental strains.

## Author contributions

AT and RP performed environmental sampling, laboratory testing and contributed significantly to the writing of the manuscript. LV and EJ performed laboratory testing, scientific advice and contributed to the writing of the manuscript. SB performed environmental sampling, laboratory testing and contributed to the writing of the manuscript. PW was responsible for study design, securing funding and contributed significantly to the writing of the manuscript.

### Conflict of interest statement

PW is a founding member of the EAPCRI, received project funding from Myconostica, Luminex, Renishaw diagnostics and Bruker, was sponsored by Myconostica, MSD, Launch, Bruker and Gilead Sciences to attend international meetings, provided consultancy for Renishaw Diagnostics Limited and is a member of the advisory board and speaker bureau for Gilead Sciences. The remaining authors declare that the research was conducted in the absence of any commercial or financial relationships that could be construed as a potential conflict of interest.

## References

[B1] Bader O. Tünnermann J. Dudakova A. Tangwattanachuleeporn M. Weig M. Groß U. (2015) Environmental isolates of azole-resistant *Aspergillus fumigatus* in Germany. Antimicrob. Agents Chemother. 59, 4356–4359. 10.1128/AAC.00100-15 25941229 PMC4468681

[B2] Bromley M. J. van Muijlwijk G. Fraczek M. G. Robson G. Verweij P. E. Denning D. W.. (2014). Occurrence of azole-resistant species of Aspergillus in the UK environment. J. Glob. Antimicrob. Res. 2, 276–279. 10.1016/j.jgar.2014.05.004 27873687

[B3] Buil J. B. van der Lee H. A. L. Rijs A. J. M. M. Zoll J. Hovestadt J. A. M. F. Melchers W. J. G.. (2017). Single-center evaluation of an agar-based screening for azole resistance in *Aspergillus fumigatus* by using VIPcheck. Antimicrob. Agents Chemother. 61:e01250-17. 10.1128/AAC.01250-17 28923874 PMC5700311

[B4] CLSI: M59-ED1 (2016). Epidemiological Cut off Values for Antifungal Susceptibility Testing. 1st Edn. Duke University Medical Centre.

[B5] Chong G. L. van de Sande W. W. Dingemans G. J. Gaajetaan G. R. Vonk A. G. Hayette M. P.. (2015) Validation of a new *Aspergillus* real-time PCR assay for direct detection of *Aspergillus* and azole resistance of *Aspergillus fumigatus* on bronchoalveolar lavage fluid. J. Clin. Microbiol. 53, 868–74. 10.1128/JCM.03216-14 25568431 PMC4390672

[B6] Chowdhary A. Kathuria S. Xu J. Sharma C. Sundar G. Singh P. K. Gaur S. N.. (2012) Clonal expansion emergence of environmental multiple-triazole-resistant *Aspergillus fumigatus* strains carrying the TR_34_/L98H mutations in the *cyp51*A gene in India. PLOS ONE 7:e52871. 10.1371/journal.pone.0052871 23285210 PMC3532406

[B7] Denning D. W. Pleuvry A. Cole D. C. (2013). Global burden of allergic bronchopulmonary aspergillosis with asthma and its complication chronic pulmonary aspergillosis in adults. *Med. Mycol*. 51, 361–370. 10.3109/13693786.2012.738312 23210682

[B8] EUCAST (2015). Clinical Breakpoints-fungi (v 8.0). Available online at: http://www.eucast.org/clinical_breakpoints

[B9] Howard S. J. Cerar D. Anderson M. J. Albarrag A. Fisher M. C. Pasqualotto A. C.. (2009). Frequency and evolution of azole resistance in *Aspergillus fumigatus* associated with treatment failure. Emerg. Infect. Dis. 15, 1068–1076. 10.3201/eid1507.090043 19624922 PMC2744247

[B10] Hurst S. F. Berkow E. L. Stevenson K. L. Litvintseva A. P. Lockhart S. R. (2017). Isolation of azole-resistant *Aspergillus fumigatus* from the environment in the south-eastern USA. J. Antimicrob. Chemo. 72, 2443–2446. 10.1093/jac/dkx168 28575384 PMC11935745

[B11] Latge J. P. (1999). *Aspergillus fumigatus* and aspergillosis. Clin. Microbiol. Rev. 12, 310–350. 10194462 10.1128/cmr.12.2.310 PMC88920

[B12] Mortensen K. L. Mellado E. Lass-Florl C. Rodriguez-Tudela J. L. Johansen H. K. Arendrup M. C. (2010). Environmental study of azole resistant *Aspergillus fumigatus* and other aspergilli in Austria, Denmark and Spain. Antimicrob. Agents Chemo. 54, 4545–4549. 10.1128/AAC.00692-10 20805399 PMC2976122

[B13] Patterson T. F. Thompson G. R. III. Denning D. W. Fishman J. A. Hadley S.. (2016). Treatment of practice guidelines for the diagnosis and management of aspergillosis: update by the infectious diseases society of America. Clin. Infect. Dis. 63, e1–e60. 10.1093/cid/ciw326 27365388 PMC4967602

[B14] Prigitano A. Venier V. Cogliati M. De Lorenzis G. Esposto M. C. Tortorano A. M. (2014). Azole-resistant *Aspergillus fumigatus* in the environment of northern Italy, May 2011 to June 2012. Euro. Survell. 19, 1–7. 10.2807/1560-7917.ES2014.19.12.20747 24698139

[B15] Risk assessment on the impact of environmental usage of triazoles on the development and spread of resistance to medical triazoles in *Aspergillus* species (2013). European Centre for Disease Prevention and Control, Stockholm: ECDC Technical Report.

[B16] Snelders E. Huis I. Veld R. A. Rijs A. J. Kema G. H. Melchers W. J.. (2009). Possible environmental origin of resistance of *Aspergillus fumigatus* to medical triazoles. Appl. Environ. Microbiol. 75, 4053–4057. 10.1128/AEM.00231-09 19376899 PMC2698372

[B17] Snelders E. Melchers W. J. Verweij P. E. (2011). Azole resistance in *Aspergillus fumigatus*: a new challenge in the management of invasive aspergillosis. Future Microbiol. 6, 335–347. 10.2217/fmb.11.4 21449843

[B18] Stensvold C. R. Jorgensen L. N. Arendrup M. C. (2012). Azole-resistant invasive *Aspergillus*: relationship to agriculture. Curr. Fungal Infect. Rep. 6, 178–191. 10.1007/s12281-012-0097-7

[B19] Tangwattanachuleeporn M. Minarin N. Saichan S. Sermsri P. Mitkornburee R. Groß U. (2017) Prevalence of azole-resistant *Aspergillus fumigatus* in the environment of Thailand. Med. Mycol. 55, 429–435. 10.1093/mmy/myw090 27664994

[B20] Van der Linden J. W. M. Arendrup M. C. Van der Lee H. A. L. Melchers W. J. G. Verweij P. E. (2009). Azole containing agar plates as a screening tool for azole resistance of *Aspergillus fumigatus*. Mycoses 52, 19–28.

[B21] Verweij P. E. Ananda-Rajah M. Andes D. Arendrup M. C. Brüggemann R. J. Chowdhary A.. (2015b). International expert opinion on the management of infection caused by azole-resistant *Aspergillus fumigatus*. Drug Res. Updates 21–22, 30–40. 10.1016/j.drup.2015.08.001 26282594

[B22] Verweij P. E. Chowdhary A. Melchers W. J. G. Meis J. F. (2015a). Azole Resistance in *Aspergillus fumigatus*: can we retain the clinical use of mold-active antifungal azoles? Clin. Infect. Dis. 3, 1–7. 10.1093/cid/civ885 PMC4706635 26486705

[B23] Verweij P. E. Snelders E. Kema G. H. Mellado E. Melchers W. J. (2009). Azole resistance in *Aspergillus fumigatus*: a side-effect of environmental fungicide use? Lancet Infect. Dis. 9, 789–795. 10.1016/S1473-3099(09)70265-8 19926038

